# Dyadic inter-brain EEG coherence induced by interoceptive hyperscanning

**DOI:** 10.1038/s41598-023-31494-y

**Published:** 2023-03-16

**Authors:** Michela Balconi, Laura Angioletti

**Affiliations:** 1 International Research Center for Cognitive Applied Neuroscience (IrcCAN), Università Cattolica del Sacro Cuore, Milan, Italy; 2 Research Unit in Affective and Social Neuroscience, Department of Psychology, Università Cattolica del Sacro Cuore, Largo Gemelli, 1, 20123 Milan, Italy

**Keywords:** Cognitive neuroscience, Attention, Neuroscience, Social neuroscience, Cooperation

## Abstract

Previous single-brain studies suggested interoception plays a role in interpersonal synchronization. The aim of the present study was to assess the electrophysiological intersubject coherence through electrophysiological (EEG) hyperscanning recording during simple dyadic synchronization tasks when the participants focused on their breath. To this aim, the neural activity of 15 dyads of participants was collected during the execution of a cognitive and motor synchronization task in two distinct IA conditions: focus and no focus on the breath condition. Individuals’ EEG frequency bands were recorded through EEG hyperscanning and coherence analysis was performed. Results showed greater EEG coherence was observed for the alpha band in frontopolar brain regions (Fp1, Fp2) and also in central brain regions (C3, C4) within the dyads, during the focus on the breath condition for the motor compared to the cognitive synchronization task; during the same experimental condition, delta and theta band showed augmented inter-individual coherence in the frontal region (Fz) and central areas (C3, C4). To conclude, the current hyperscanning study highlights how the manipulation of the interoceptive focus (obtained through the focus on the breath) strengthens the manifestation of the EEG markers of interpersonal tuning during a motor synchronization task in specific brain areas.

## Introduction

Interoception has been classically defined as the mechanism through which our brain perceives and integrates the information derived from our body^1^. Current definitions suggest that interoception is not a static type of perception, but a dynamic one, which can be modified and trained, for instance, by mind-and-body awareness practices. In particular, among the different components of interoception, “the attention to one’s inner body signal (such as the heartbeat or the breath) for a given interval of time” (i.e., Interoceptive Attentiveness, IA^2,3^) can be manipulated, modulated, and trained to impact positively on the individual’s emotional and cognitive well-being^4^. This high order ability to intentionally focus on one’s body signal is the opposite of the exteroceptive attention, that is the attention to stimuli arising outside of the body.

Previously, interoception has been mainly studied as a process connected to the internal world of the individual, only recently new studies are focusing on how interception has an impact on the external world. To this regard, the term “social interoception” was introduced in the literature to refer to how interoception affects a variety of social processes, including self-other distinction^5^, social cognition (conceptualized in terms of the fundamental elements of theory of mind, empathy, and imitation^6^), social isolation and connectedness^7^ and emotional experience^8–10^. According to a developmental perspective, infants build expectations about the cause of their internal sensations via a dynamic process of interoceptive distinction between self and other, and develop their interoceptive processing through a fundamental social process, that is the caregiver-infant feeding interaction^11^, but also through social touch^8^. Research on the psychophysiological coupling between caregivers and infants^12^ is investigating the social origins of interoception^13,14^ and suggested that caregivers play a key role in both detecting the infant’s interoceptive perturbations that result in behavioral expressions of affective feelings as well as in providing a proper response to support the regulation of the infant’s needs. The infant's brain will gradually begin to mentalize her own interoceptive experiences and eventually conduct appropriate behaviors through such embodied interactions^13^. Thus, it could be relevant to study interoception in a social-interactional setting for deepening how humans’ interpersonal synchronization is mediated not only by the central nervous system and higher order processes, but also by lower-level functions, such as motor synchronization induced by respiration, which have a direct effect on high-order social processes.

Nonetheless, so far, there have only been a few research efforts, particularly in the neuroscientific domain, looking at how interoception manipulation might affect the process of interpersonal synchronization. Before, single brain studies were conducted to explore the hemodynamic and electrophysiological (EEG) neural correlates of the IA manipulation (conceived as the focus on the breath for a given time interval) on interpersonal synchronization required by performing simple motor or cognitive joint tasks^15–18^.

Starting from the hemodynamic correlates of IA manipulation on interpersonal synchronization, for instance, functional Near-Infrared Spectroscopy (fNIRS) was exploited to record the oxygenated haemoglobin (O_2_Hb) changes during joint tasks involving motor and cognitive synchronization while participants were required to focus their attention on the breath^15,17^. Hemodynamic results suggested that the whole prefrontal cortex (PFC), which is involved both in sustained attention, reorientation of attention, social responsiveness, and synchronization, was more responsive when inducing the explicit focus on the breath (explicit IA condition) during a socially framed motor task requiring synchronization (as indicated by increased O_2_Hb): in the absence of a broader and explicit social frame, this effect was not significant for the motor task^17^. Additionally, when an explicit focus on the breath was induced during the cognitive synchronization task, a hemispheric lateralization was suggested with an increase of O_2_Hb in the right PFC^15^.

Also, recent contributions examined the link between interoception, respiration and neural oscillations recorded by EEG^19–21^. Some works suggested that the conscious control of respiration rhythms (e.g., slow-paced or nasal respiration) influence oscillatory entrainment^22,23^. Specifically, slow breathing seems to modulate the alpha band range across the temporal and prefrontal lobes^22^, while respiration-entrained oscillations may modulate local gamma activity^23^. However, other authors^24^ did not find an impact of respiration rate changes on intracranial EEG-breath coherence. This last evidence may be due to the fact that this effect was related to the impact of attention to breath, which is different from volitional breathing.

Other studies provided support for the hypothesis that breathing rhythmically can lead to intra-individual neural synchronization through oscillations^20,25^, while it has not yet been clarified whether this may have an effect on interpersonal neural synchronization. Interestingly, Tschacher and Meier^26^ observed that interpersonal physiological synchrony develops between clients and psychotherapists, particularly for breathing, and that this physiological linkage is positively correlated with client alliance and the therapist assessment of the progress of a therapeutic session; however, the authors did not test the impact of breathing synchrony on interpersonal neural synchronization. Instead, Coomans and colleagues^27^ observed interpersonal neural synchrony for theta and alpha bands (i.e., intersubject EEG coherence) while healthy dyads were practising a mindful breathing exercise (without controlling the respiratory rate or synchrony). Thus, it might be plausible that entrainment through external rhythms (e.g., two people breathing in synchrony or engaged in an attention-to-breath exercise) could lead to interpersonal neural synchrony during dyadic interactions. The interrelation between physiological (e.g., respiration) and neural synchrony have most often been studied in isolation for issues of measurement timing^28^. Therefore, future studies are needed to deepen if dyads breathing in synchrony or directing the focus of attention on the process of spontaneous breathing display EEG interpersonal neural synchrony.

With regard to single-brain EEG evidence on IA and synchronization, in a first recent work, EEG frequency bands (delta, theta, alpha, and beta bands) were acquired from the frontal, temporo-central, and parieto-occipital regions of interest (ROIs) while neurotypical participants performed a motor and a cognitive synchronization task during the focus and no-focus breath conditions^18^. Results showed two main patterns of frequency band modulation during the execution of a motor compared with the cognitive synchronization task while a person is focusing the attention on one’s breath: first, a significantly higher delta and theta power in the focus on the breath condition in the frontal region during the execution of the motor than the cognitive synchronization task; and, second, in the same experimental condition, delta and beta band power increased in the temporo-central area.

In a previous EEG hyperscanning study, delta and theta band synchronization within and between guitar players was enhanced at the frontal and central electrodes during periods requiring high demand on musical coordination^29^. Moreover, delta and theta bands in the frontal regions were observed during meditative practice^30^ and a focused meditative state^31^, two conditions that require but also strengthen the sustained interoceptive focus on breath sensations^1,32^. Stronger delta activity in the prefrontal cortex was previously related to inhibitory function and detachment during meditation^30^. Increased frontal theta was observed in previous EEG studies of breath-focused meditation^33,34^ and may indicate a need for cognitive control^35^. Also, the manifestation of beta oscillations was observed during behavioral motor synchrony in distinct regions of the brain^36^, including the centro-parietal areas^37^. Beta band was previously related to sensorimotor activity but also to higher-order processes such as prediction^38^, confidence^39^, and gain control^40^. This evidence suggested that interoception (conceived as the focused attention on the breath) improves the manifestation of EEG brain correlates related to mental concentration, coordinated, and controlled motor activity especially during motor synchronization activities.

In a more recent EEG study, the same experimental procedure was adopted but both tasks were socially framed^16^. Indeed, to stress the shared intentionality and increase ecological validity, both tasks were socially framed by specifying to the participants that they need to synchronize during these tasks to develop greater teamwork skills. In this work, an increase in delta band and desynchronization of alpha band (EEG delta-alpha pattern) emerged in the temporo-central areas at the intra-individual level, indicating the attention to visceral signals, particularly during interpersonal motor synchrony compared to the cognitive synchronization task. This evidence was interpreted considering the functional meaning of delta and alpha band in relation to motor synchronization. Delta motor oscillations reflect the dynamics of motor behaviors and motor neural processes^41^, whereas alpha band attenuation, which has previously been observed during the creation, observation, and visualization of movement and is thought to reflect cortical motor activity and action-perception coupling^42,43^.

Overall, findings suggested that interpersonal motor synchronization occurs in specific areas (i.e., in the frontal PFC and in temporo-central areas) and that this synchronization is amplified and mainly involves low frequency bands (with an increase of delta and decrease of alpha band), which reflect a synchronous trend probably produced by the focus on breathing (as partially suggested by studies on meditation^30^).

Despite the fact that these studies were the first observing the effect of IA manipulation on single individuals performing simple motor and cognitive tasks in synchrony with another partner and helped in determining the neurofunctional basis and the EEG correlates of this phenomenon at the intraindividual level, one evident limitation consisted in the lack of measures of the interactional dynamics between the two members of the dyad. In fact, with the advent of hyperscanning paradigm^44^, different studies computed inter-agent synchronization and inter-brain coupling metrics mirroring the level of social attunement based on simultaneous recording of behavioral and electrophysiological responses from different agents involved in a joint task or a social exchange^45,46^ and this also allowed to explore the inter-individual neural synchronization of dyads while performing motor and cognitive joint tasks in cooperative or competitive conditions (for a review see the contribution of Balconi and Vanutelli^47^).

For instance, with regard to motor tasks, in the study of Novembre and colleagues^48^, short musical pieces were played by piano duos while behavioral synchronization was manipulated: high behavioral synchrony was associated with a decrease of alpha power in the right centro-parietal region (conversely, low behavioral synchronization with an increase of alpha power). About cognitive synchronization, by using the hyperscanning paradigm also live face-to-face interactive speech has been investigated (for a review see the work of Kelsen and colleagues^49^). However, these hyperscanning studies did not manipulate the interoceptive focus while performing the joint tasks.

Therefore, the primary aim of the present study was to assess electrophysiological intersubject coherence through EEG hyperscanning recording during simple dyadic synchronization tasks performed in two distinct interoceptive conditions. The experimental design examined two distinct conditions of presence and absence of interoceptive focus (that is when the attention of the participants was focused on the breath versus not focused on the breath), as well as the specific synchronization task performed by the participants (cognitive versus motor).

By moving towards a two-person neuroscience approach, this study exploited neural coherence indices to investigate the EEG frequency bands of between-brain connectivity. Coherence indices were previously adopted in EEG hyperscanning studies to explore brain rhythm synchronization during cooperative and competitive joint actions^50^, real-life conversations in the work context, such as a performance interview^51,52^ and a job assessment interview^53,54^.

Given previous evidence, we hypothesized to observe higher EEG coherence during the focus on the breath condition mainly in the motor compared to the cognitive synchronization task, since the motor synchronization task was previously shown to be more sensitive to the interoceptive manipulation^17^, perhaps due to the proximity between motor and interoceptive areas.

Secondly, during the focus on the breath condition in the motor compared to the cognitive synchronization task, we expected to observe this inter-brain coherence effect for specific frequency bands, namely low frequency bands, such as delta, theta, and alpha, given their twofold role in sustained attention and focus on the meditative state^16,31,55^, and controlled motor synchronization^56^. Also, during the same experimental condition, an increase in beta band coherence, as a marker of the sensorimotor system propensity to uphold the status quo^57^, is expected.

Thirdly, as indicated in the literature reported above, we hypothesized that the inter-brain coherence effect due to this interoceptive focus manipulation will involve mainly frontal and temporo-central electrode locations in the brain, in line with the previous studies highlighting these regions, as the areas connected to attentional control and inter-brain phase synchrony, whose EEG responsiveness is solicited by the interoceptive manipulation^16,18^.

**Figure 1:**
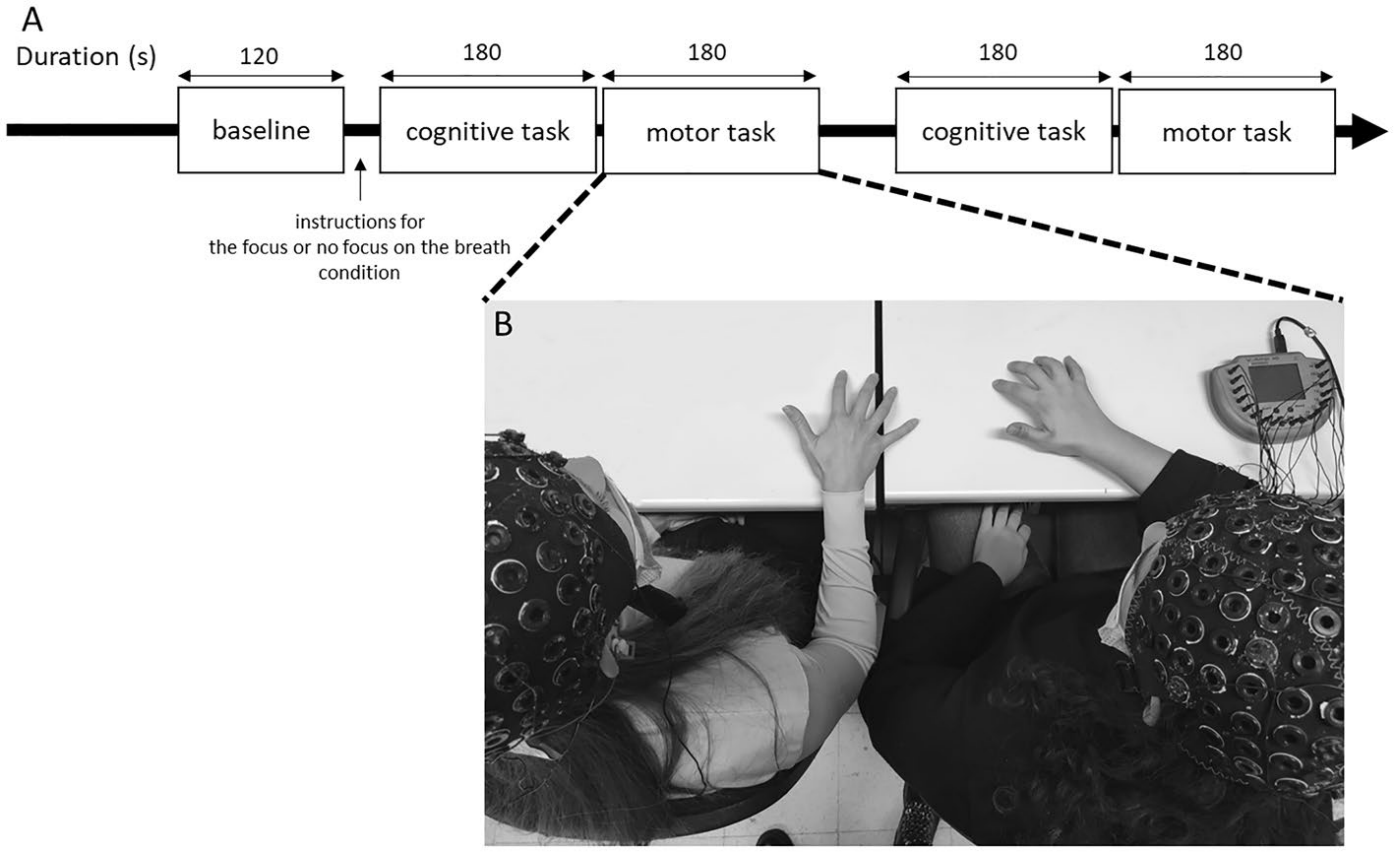
(**A**–**B**) Experimental procedure. Experimental procedure representing the setting for the joint task and the EEG hyperscanning acquisition from the dyad. To avoid order effect, the task execution was randomized and counterbalanced for the type of the task and the condition.

Finally, considering that coherence indices were previously adopted in EEG hyperscanning studies to explore brain rhythm synchronization, we aim to test if the inter-subject EEG coherence indices can be considered as a valid marker of inter-individual dyads synchronization when the interoceptive focus on the breath is manipulated (Fig. 1).

## Results

### First step: Coherence analysis

**Figure 2:**
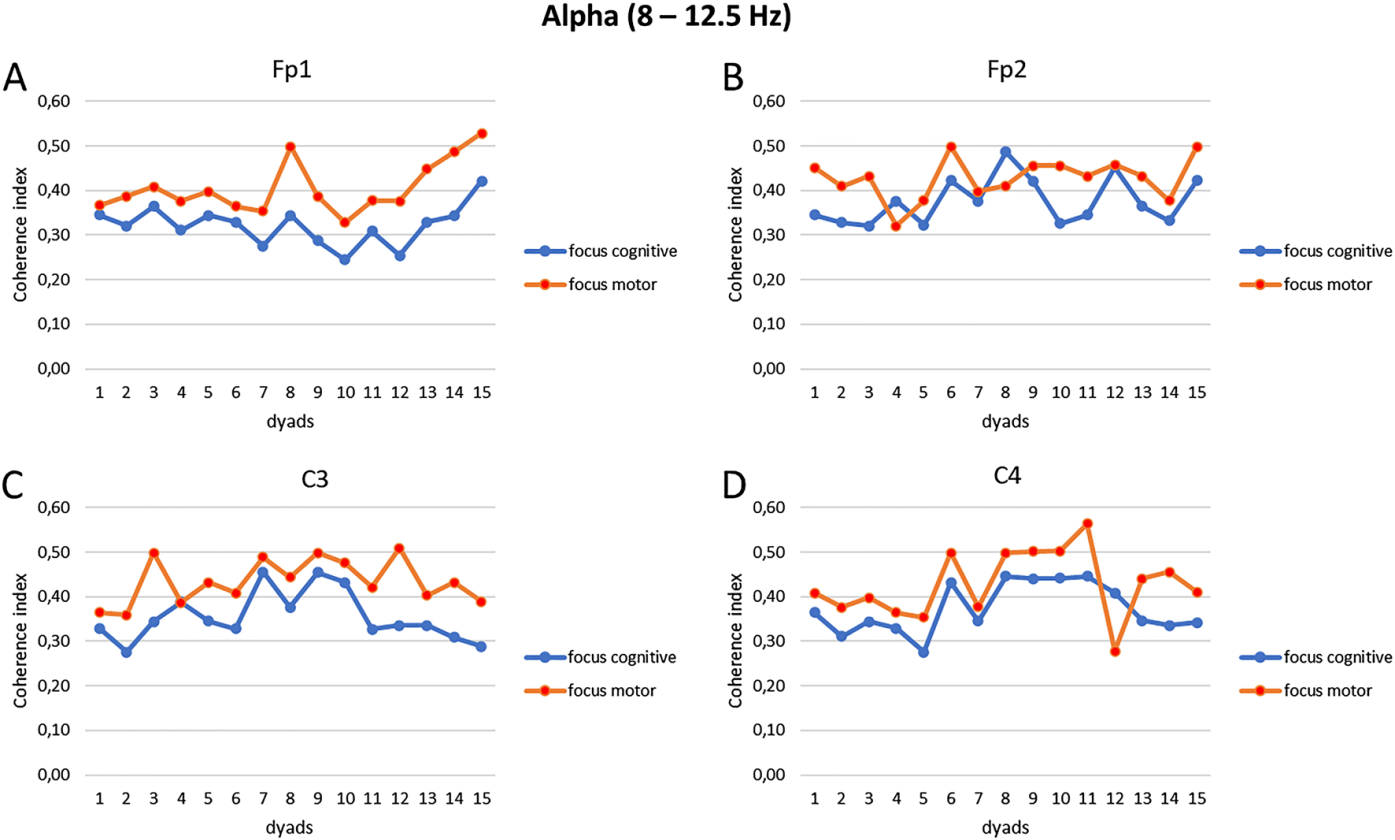
(**A**–**D**) EEG coherence indices for alpha band. Trend of the coherence indices modulation as a function of the synchronization tasks for the electrodes Fp1 (**A**), Fp2 (**B**), C3 (**C**), and C4 (**D**) in each dyad.

**Figure 3:**
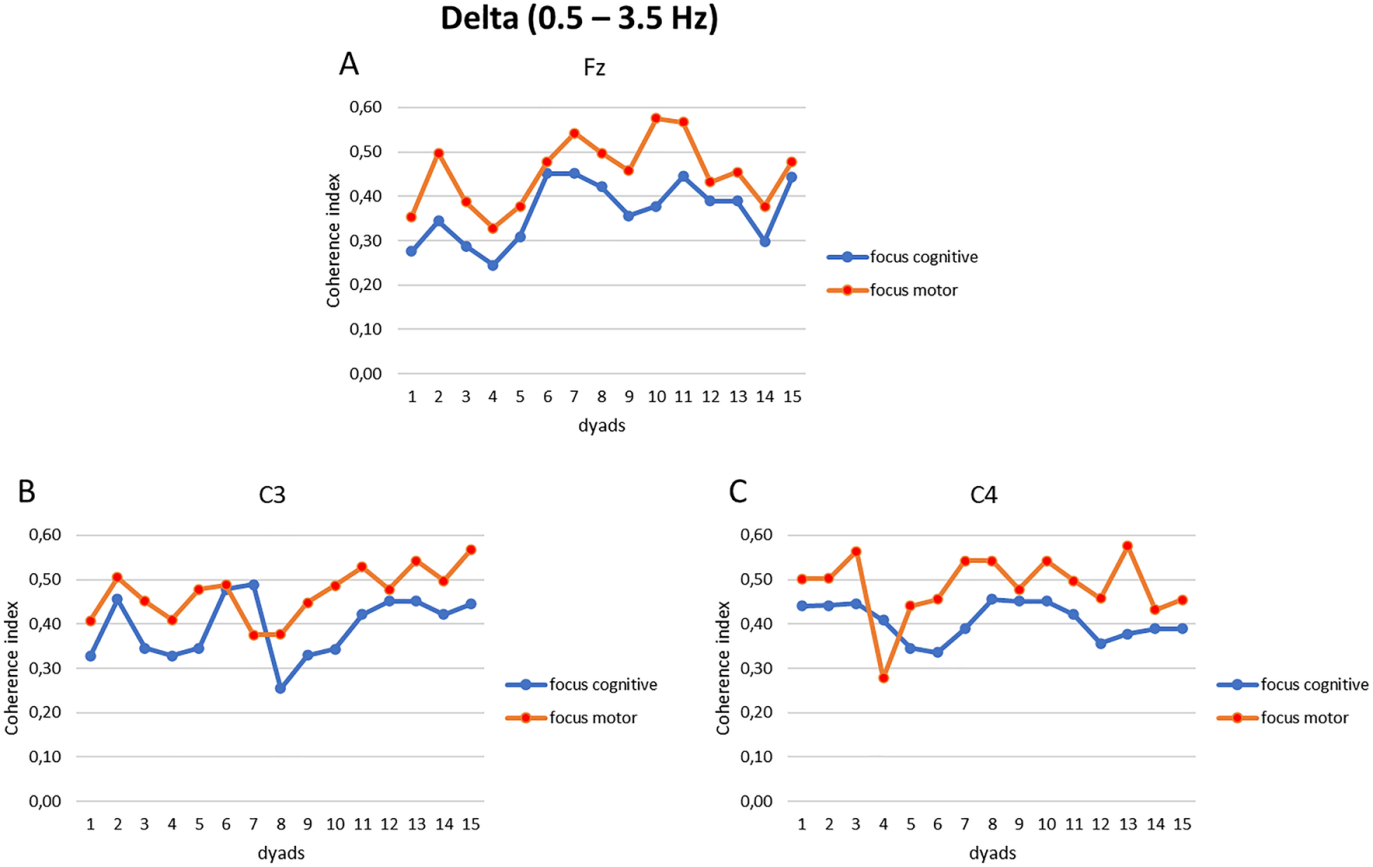
(**A**–**C**) EEG delta band coherence indices. Trend of the coherence indices modulation as a function of the synchronization tasks for the electrodes Fz (**A**), C3 (**B**), and C4 (**C**) in each dyad.

**Figure 4:**
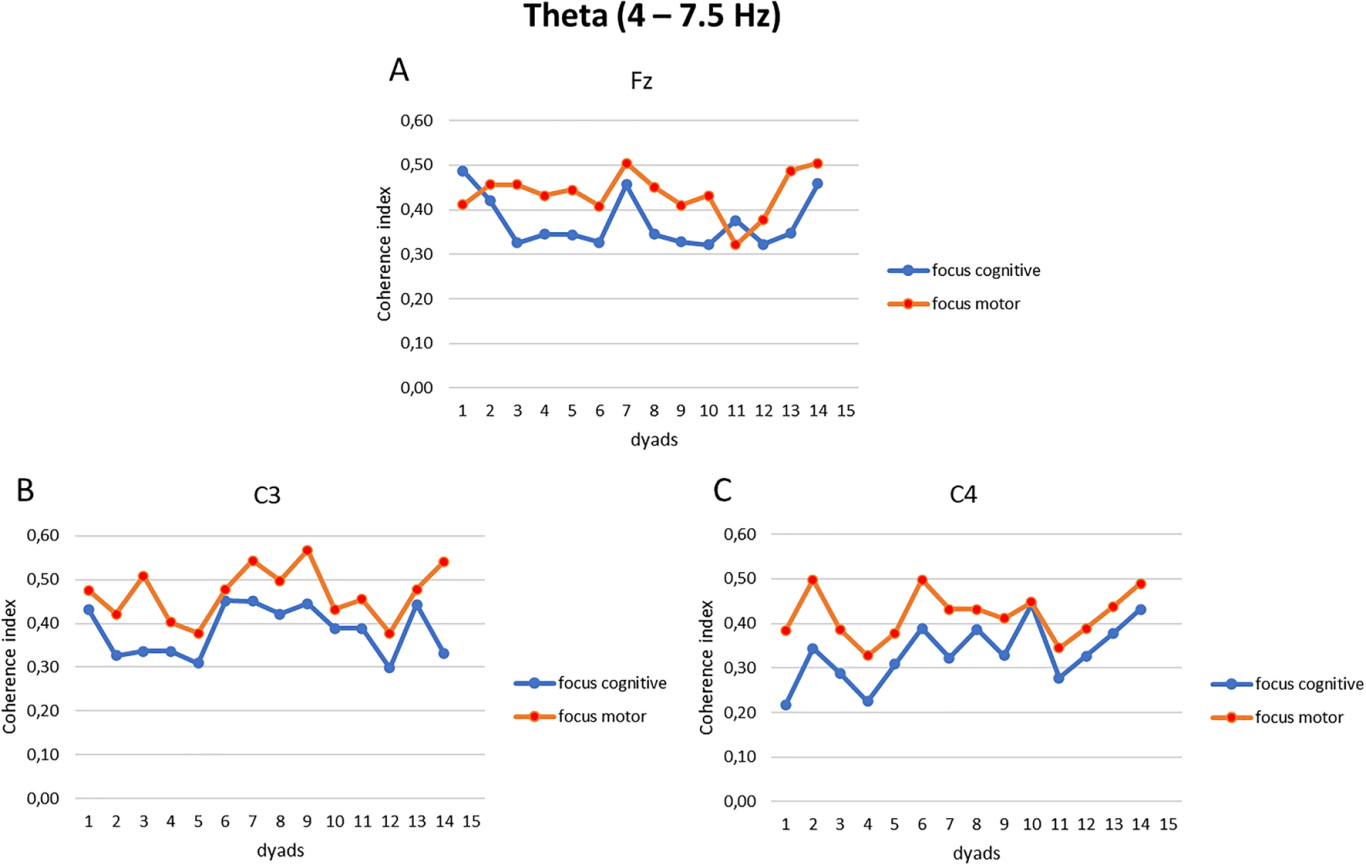
(**A**–**C**) EEG theta band coherence indices. Trend of the coherence indices modulation as a function of the synchronization tasks for the electrodes Fz (**A**), C3 (**B**), and C4 (**C**) in each dyad.

As result of the first step of analysis, we have reported below the computed coherence values related to a selected number of electrodes (which were significant at the successive ANOVAs) for each frequency band: alpha (Fig. 2A–D), delta (Fig. 3A–C) and theta (Fig. 4A–C).

### Second step: ANOVAs

Since all experimental conditions differed from the baseline, a successive analysis was conducted to compare only the experimental conditions as independent factors. The ANOVA applied to inter-brain coherence indices as dependent variables for each dyad revealed significant effects for alpha, delta and theta frequency bands. The following paragraphs report the significant results obtained for the ANOVAs.

#### Alpha

Indeed, for alpha band, first significant interaction effect was observed for Condition × Electrode (F[14, 14] = 8.77, *p* = 0.01, η^2^ = 0.349). Pairwise comparisons revealed an increase in coherence within the dyads during the focus on the breath compared to the no focus on the breath condition in the following electrodes Fp1 (F[1, 14] = 5.67, *p* = 0.01, η^2^ = 0.278), Fp2 (F[1, 14] = 4.43, *p* = 0.05, η^2^ = 0.227), C3 (F[1, 14] = 4.89, *p* = 0.05, η^2^ = 0.290), and C4 (F[1, 14] = 6.76, *p* = 0.01, η^2^ = 0.408).

**Figure 5:**
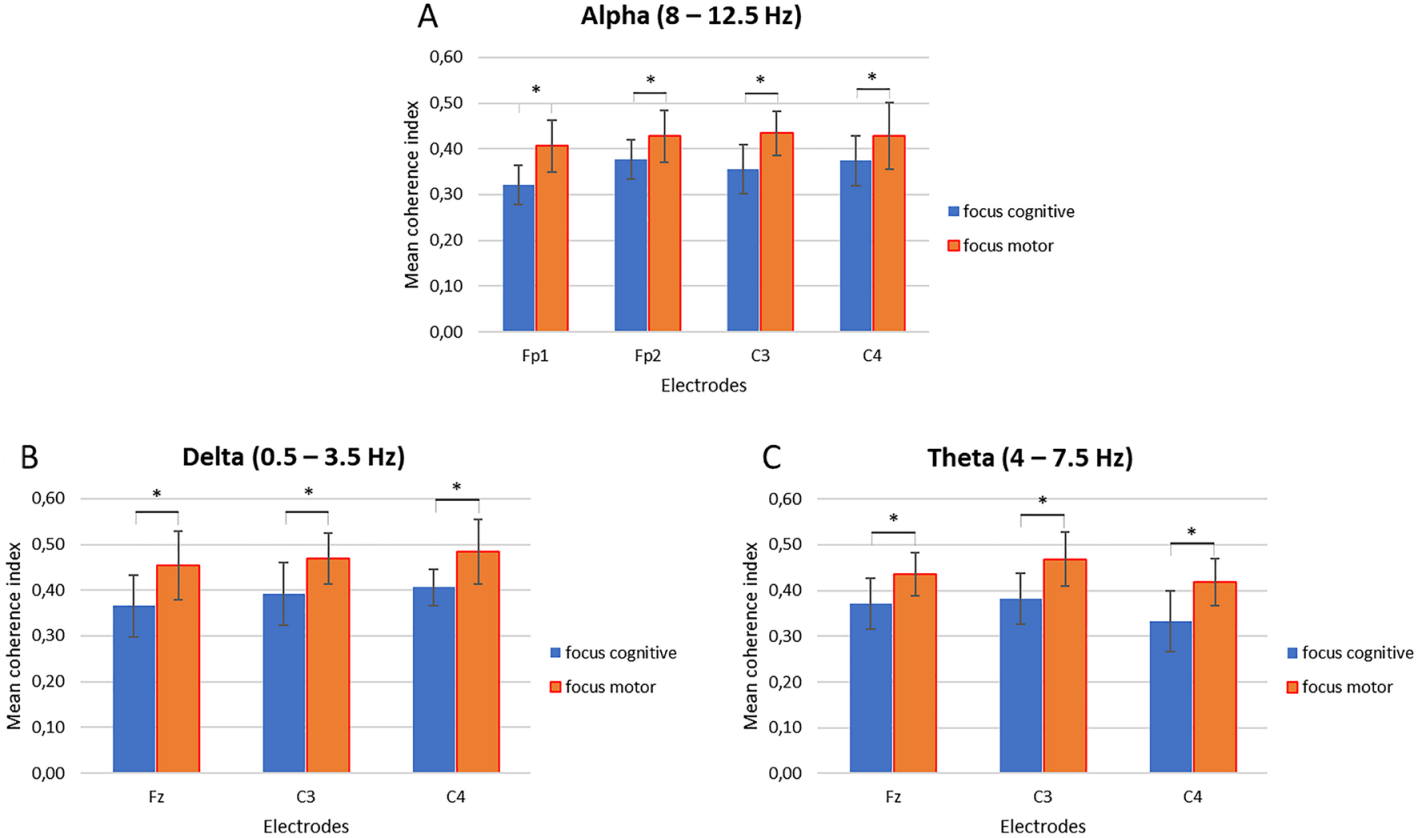
(**A**–**C**) Mean coherence indices for alpha, delta, and theta frequency bands. Bar graphs show the mean values of coherence indices (± SE) for the alpha (**A**), delta (**B**), and theta (**C**) band under the focus condition during the motor compared to the cognitive synchronization task. All asterisks (*) mark statistically significant differences, with *p* ≤ 0.05.

Secondly, a significant interaction effect was observed for Condition × Task × Electrode (F[14, 14] = 7.89, *p* = 0.01, η^2^ = 0.499). In particular, pairwise comparisons showed greater coherence within the dyads during the focus on the breath condition for the motor compared to the cognitive task in the following electrodes Fp1 (F[1, 14] = 5.09, *p* = 0.01, η^2^ = 0.378), Fp2 (F[1, 14] = 5.89, *p* = 0.01, η^2^ = 0.390), C3 (F[1, 14] = 6.09, *p* = 0.01, η^2^ = 0.411), and C4 (F[1, 14] = 7.09, *p* = 0.01, η^2^ = 0.442) (Fig. 5A). No other statistically significant effects were found.

#### Delta

About delta band, the interaction effect of Condition × Task × Electrode displayed significant differences (F[14, 14] = 6.09, *p* = 0.01, η^2^ = 0.451). Pairwise comparisons showed augmented coherence within the dyads during the focus on the breath condition for the motor compared to the cognitive task in the following electrodes Fz (F[1, 14] = 6.09, *p* = 0.01, η^2^ = 0.378), C3 (F[1, 14] = 7.08, *p* = 0.01, η^2^ = 0.421), and C4 (F[1, 14] = 5.08, *p* = 0.01, η^2^ = 0.387) (Fig. 5B). No other statistically significant effects were found.

#### Theta

For theta band, the interaction effect of Condition × Task × Electrode displayed significant differences (F[13, 14] = 5.90, *p* = 0.01, η^2^ = 0.370). Pairwise comparisons showed augmented coherence within the dyads during the focus on the breath condition for the motor compared to the cognitive task in the following electrodes Fz (F[1, 13] = 6.01, *p* = 0.01, η^2^ = 0.329), C3 (F[1, 13] = 4.99, *p* = 0.05, η^2^ = 0.289), and C4 (F[1, 13] = 5.98, *p* = 0.01, η^2^ = 0.387) (Fig. 5C). No other effect was statistically significant.

## Discussion

The current study aimed at investigating and assessing the markers of interpersonal tuning of neurotypical participants during simple dyadic synchronization tasks (motor- and cognitive-based) performed in two distinct interoceptive conditions, that is when the attention of the participants was focused on their breath versus not focused on their breath. A social neuroscientific hyperscanning approach by EEG was applied to allow the recording of participants’ eletrophysiological responses related to the motor and cognitive synchronization tasks. For the EEG signal, we performed analyses of the coherence indices and a comparison of EEG coherence’s strength for the conditions, tasks and electrodes.

First, a coherence analysis was computed to check the inter-subject EEG coherence between the dyads for each EEG electrode considered in each experimental condition; it was chosen to report the main statistically significant results in graphs to describe the trend of synchronization of the dyads. Secondly, some relevant and significant findings were observed: greater EEG coherence was observed for alpha band in frontopolar brain regions (Fp1 and Fp2) and in central brain regions (C3 and C4) within the dyads, during the focus on the breath condition for the motor compared to the cognitive synchronization task; during the same experimental condition, delta and theta band showed augmented inter-individual coherence in frontal region (Fz) and central areas (C3 and C4).

According to the first hypothesis, the findings of the present study displayed higher EEG coherence, in terms of dyadic tuning, during the interoceptive focus on the breath condition when participants where performing the motor compared to cognitive synchronization task. We may suggest that the increase of coherence during the focus on the breath condition demonstrates the role of the interoceptive focus as a generator and activator of synchronization, as a dynamic process that also requires interpersonal tuning at the neural level. In the current work, findings confirmed former evidence^18^ and showed that this neural synchronization becomes more evident during the motor compared to the cognitive synchronization task, perhaps because of the neuroanatomical proximity between interoceptive networks and sensorimotor areas, or alternatively because of a link between breathing and motor synchronization. Indeed, previous studies stated that the breath plays a special role in mediating respiration-entrained brain synchrony enhancing motor activity^58^ and synchrony in the motor cortex^24^. However, for the first time, this interoceptive effect on motor synchronization (as a condition promoting full synergy, if compared to the cognitive-based task) has been confirmed by observing the EEG neural synchronization during a real interactive dynamic of two individuals, through hyperscanning paradigm.

Partially in line with our second hypothesis, this neural synchronization was mainly observed for low frequency bands, that is alpha, delta, and theta bands. With reference to alpha band, Pérez and colleagues^59^ found that speakers in the central region and listeners in the frontal area showed neuronal alpha synchronization. More recently, Coomans and colleagues^27^ assessed intersubject EEG bands’ coherence of dyads performing individual and joint practice of mindful breathing exercise. Authors observed an increase of EEG coherence in the joint practice session compared to the individual session in frontal and temporal regions for alpha band, interpreted as an increased shared relaxation, and in temporal regions for theta band, associated to the agreeableness of the dyad and to theory of mind. Also, it has been suggested^60^ that enhanced alpha synchronization over frontal regions (Fp1 and FP2) reflects “switching off” mechanisms of external attention. Beauregard and colleagues^61^ stated that higher alpha power detected over frontal (Fp1 and Fp2) and left temporal regions during meditation condition is an index of reduced cortical arousal associated with a relaxation response.

For what concern the significance of delta and theta band in motor synchronization tasks, prior studies have shown that when playing a brief melody, guitarist pairs exhibit more synchronized theta and delta oscillations in frontal and central electrode sites. This may be because of coordinated firing of neuronal assemblies in the motor and somatosensory cortex, which orchestrate and regulate motor activity, as well as frontal regions supporting social cognition^56^. Also, the frontal midline theta rhythm was associated with the parasympathetic component of the autonomic nervous system^62–64^. Significant increases of theta power in frontal midline electrodes Fz, FCz, and Cz (anterior cingulate source) was observed after five days of integrative body–mind training^65^.

A possible explanation is that our results suggest a low band tuning due to greater cooperation induced by a simple synchronization task, where, in particular, the motor task highlights the real synchrony effect. The interoceptive focus is effective on the low frequency bands and it also promotes a motor synchrony, whereby the effect of the attention on the breath acts directly on the motor synchronization. Since the control condition (without the focus on the breath) during the motor task does not yield the same effect, it is arguable that is precisely the interoceptive focus linked to this effect. Moreover, it appears that the central brain locations (C3 and C4) are the sites where the interoceptive focus has the highest expression; however, it has to be tested in future studies whether the same effect occurs with different motor synchronization tasks.

Differently from what was expected, no significant effects, in terms of EEG coherence indices, were instead observed for beta band. Several studies demonstrated beta rhythms desynchronize before and during a movement and resynchronize after task completion in sensorimotor cortex^66–69^.

Despite a beta band increase in the temporo-central region when performing the motor compared to cognitive synchronization task during the focus on the breath condition was observed in a previous single-brain study^18^, a possible explanation for the absence of this effect could be that beta may not be a key neural marker of breath-based interoceptive manipulation in motor synchronization tasks. However, given the role of the beta band in sensorimotor synchronization, this evidence needs to be eventually confirmed by future studies.

Moreover, no significant results were detected for the focus on the breath condition while the participants were performing the cognitive synchronization task. This lack of statistically significant results may be explained by the complexity of the task, which called for the use of various cognitive processes. In fact, a modified form of the human-to-human alternating speech task was used for the current investigation, in which participants had to syllabify in synchrony for a total of 3 min. A possible alternative reason for this absence of results could be that the verbal register’s mediation has made it difficult for people to focus on their breathing and coordinate it with their speech, which may have increased their cognitive load and necessitated the activation of a more diverse and scattered neural network (such as, both the frontal and the temporo-parietal areas^49^).

To conclude, the current hyperscanning study highlights how the manipulation of the interoceptive focus (obtained through the focus on the breath) strengthens the manifestation of the EEG markers of interpersonal tuning during a motor synchronization task. This may be of interest to basic neuroscientific research, indeed to the best of our knowledge this is the first time that the influence of interoception in an interactive social dynamic involving two people is investigated, as a true expression of this what we mean by “social interception”. We believe that interoception can affect social processes, in terms of increased social empathy and tuning for others’ actions. In this case, for motor activity, it is possible that it is increased by the synchronization of other motor action, with a distinct nature (respiration could be considered as a form of motor control on automatic processes), mediated by attentive processes. The respiration and the synchronized finger tapping can be considered as two motor activities that, synchronized by attentional focus, may reinforce each other.

Furthermore, these findings could be useful for athletes and sports coaches, to implement intervention protocols based on these neural markers (e.g., bio/neurofeedback interventions)^70^, for mind-and-body therapies dedicated to typical and clinical samples^71,72^, and/or for neuromotor rehabilitation professionals to map the progress derived from focusing on the breath during synchronized motor exercises, or promote effects in which the focus on the breath drives the motor synchronization to rehabilitate its functioning.

Despite the novelty of this study, we can highlight some weaknesses that could be addressed in future studies. For instance, a more comparable cognitive synchronization task, in terms of easiness of execution, could be adopted in prospective research to properly check the effect of IA manipulation on this cognitive activity. There is also a lack of comparison of the EEG data during the baseline and task conditions, which may be recommended as a control analysis in future studies. Furthermore, the integration of other neuroscientific tools (such as magnetoencephalography^73^) and techniques (such as, the adjunctive analyses of other EEG frequency ranges; 3.5–4 Hz; 7.5–8 Hz; 12.5–13 Hz) could contribute to the study of the electrophysiological correlates underlying the mechanisms that characterize the influence of focus on one’s body during a simple social interaction. In fact, another limitation concerns the low number of electrodes considered and the lack of source localization, that would not allow the study to make a very strong inference on the locations/brain areas related to the observed effects. Cautious conclusions must be drawn from this study since, in addition to exploiting an EEG montage with a relatively small number of electrodes, the significant results were only observed on a few channels (i.e., Fp1, Fp2, Fz, C3, and C4).

Moreover, in the context of this study, it might be that sitting next to another person could lead to focusing on the other’s breathing (as well) and have an effect on brain activity besides interoceptive attention on one’s own breathing^74^, and this experimental condition should be controlled in further research. Also, the lack of respiration measures (performed with a respiration belt or videorecording) would promote the control of the voluntary component of the breath in the future. Further studies could also consider controlling any respiratory infections, allergies or asthma that could influence attention to the breath.

Finally, a relatively low sample size was used in the study and no power analysis was conducted in the absence of a population that could serve as a reference sample. Therefore, a larger sample size might be recommended for further studies in order to better generalize the current results.

## Method

### Sample

Through a non-probabilistic convenience sampling method, a total of 30 university students were recruited for the current EEG experiment (14 females; age Mean = 24.8; Standard Deviation = 3.38) and were randomly paired in dyads matched for gender and age. Pregnancy, prior meditative experience, severe physical and chronic diseases, convulsions, chronic pain, and any mental or neurological abnormalities were among the physiological criteria of exclusion. All participants were right-handed and had normal or corrected-to-normal vision. They voluntarily took part in the experiment after completing and signing a written informed consent form and were informed that they would not be compensated for their participation in the study. This study was carried out in accordance with the Declaration of Helsinki and approved by the Ethics Committee of the Department of Psychology, Catholic University of the Sacred Heart, Milan, Italy (Approval code: 2020 TD—for thesis dissertation; approval date: 20–21).

### Procedure and interoceptive manipulation task

Each dyad was seated next to each other, in a way that they could easily interact face to face. Before commencing the experiment, procedural instructions were given to the participants. They were told they would be asked to perform two joint synchronization tasks in two distinct experimental conditions in which the IA was manipulated. In the first condition, IA was explicitly manipulated by asking the individuals to focus their attention on their breath. In this focus on the breath condition, the following instructions were provided: “During this task, we ask you to concentrate on your breathing. Try to pay attention to how you feel and whether your breathing changes as you complete the activity.” Participants were not instructed to a pace-specific breath. While, in the no focus on the breath condition, considered as the control condition in which interoception was not manipulated, no specific instruction was provided, and participants were just told to perform the joint tasks. To maintain the reliability of the procedure, the same interoceptive manipulation was adopted in previous studies and proved to affect EEG and hemodynamic neural correlates^15–18^.

Moreover, before starting with the synchronization tasks, a 120-s EEG resting baseline was collected from the two members of the dyad. To avoid potential biases related to sequence effects, the condition and the synchronization tasks’ execution order were randomized and counterbalanced. At the end of the tasks, there was a debriefing phase in which participants declared (on a scale from 0 to 10 points) the attention they paid to their breathing, to the other person and to the task (see Supplementary Material). The whole experiment lasted a total of 40 min.

### Description of the joint motor and cognitive synchronization tasks

For the motor synchronization task participants had to coordinate and synchronize their finger-tapping movement for three minutes with the other member of the dyad. Specifically, participants were asked to sit on a chair and position the fingers of their dominant hand approximately a centimeter apart, with their elbows resting on the table. They were asked to tap the table with all fingers of their dominant hand. They were not instructed to do this motion at a specific pace or to extend their fingers as far as they could. The only requirement was that they match the movement of their fingers like that of the participant sat in front of them. The average number of loops, counted as times for a whole finger-tapping sequence, was 60.

For the cognitive synchronization task, a modified form of the human-to-human alternating speech task^75^ was used, in which participants had to syllabify in synchrony for a total of 3 min with the participant of the dyad. The participants were instructed to pronounce the four syllables “LA”, “BA”, “CA”, and “DA” sequentially and alternately. For instance, when one member of the dyad says “LA”, the other member should pair the syllable by saying “LA”, and so on, in order to pronounce it at the same time. The speech patterns were not decided upon beforehand. Each language synchronization task session lasted three minutes without pauses. The average number of loops throughout the three minutes—the number of repetitions from “LA” to “DA”—was at least 45.

To promote consistency in the experimental procedure, the same tasks used in previous single-brain studies^11^ were used for this hyperscanning study (Fig. 1).

### EEG data recording and coherence analysis

EEG data acquisition was performed by adopting two 16-channel portable EEG (V-AMP: Brain Products, München; LiveAmp: Brain Products, GmbH, Gliching, Germany). Two ElectroCaps with Ag/AgCl electrodes grounded to the earlobes (10/5 system of electrode placement^76^) were applied. Electrodes were positioned over the following positions Fp1, Fp2, AFF5h, Fz, AFF6h, T7, C3, Cz, C4, T8, P3, Pz, P4, O1, O2 for both participants. Data were collected with BrainVision Recorder software (Brain Products GmbH, Munich, Germany) using a bandpass filter of 0.01–250 Hz, a sampling rate of 1000 Hz and a 50 Hz notch input filter. Prior to data collection, the recording electrodes’ impedance was examined and was consistently less than 5 kΩ. To avoid signal-to-noise ratio distortions, an off-line common average reference was utilized^77^. In order to record eye movements, an EOG electrode was also placed on the eye’s canthi.

Both resting-state and tasks-related data were filtered offline with a 0.5–45 Hz IIR filter (slope: 48 dB/octave), then segmented, and ocular inspection was applied for residual ocular, muscle, or movement artifacts (rejected epochs, 2%). To increase specificity, only artifact-free epochs were considered. EEG power spectra for artifact-free segments were finally computed via Fast Fourier Transform, averaged to calculate condition-specific power spectra, and the following frequency bands were then extracted: Delta (0.5–3.5 Hz), Theta (4–7.5 Hz), Alpha (8–12.5 Hz), and Beta (13–30 Hz). BrainVision Analyzer 2.0 (Brain Products GmbH, Munich, Germany) was employed for EEG data reduction.

For the neural synchronization, two devices with the same software were used, and the two identical systems were synchronized and supported by Brain Vision Recorder (Brain Products GmbH, Munich, Germany). For the subsequent statistical analyses, coherence analyses on the biosignal were applied as a statistical approach. Indeed, a series of analyses were conducted to obtain inter-brain connection (inter-brain coherence), by computing the partial correlation coefficient Πij for each dyad, applied to each frequency band. They were obtained by normalizing the covariance matrix's inverse

$$\Gamma = \Sigma^{{ - {1}}}$$

$$\Gamma = \left( {\Gamma_{{{\text{ij}}}} } \right) = \Sigma^{{ - {1} }} {\text{inverse}}\;{\text{of}}\;{\text{the}}\;{\text{covariance}}\;{\text{matrix}}$$

In comparison with previous approaches, such analysis enables assessing the link between two signals (i, j) independently of one another^78^, while taking into account some targeted frequency bands of interest and their functional significance. Furthermore, this approach has been often used in previous EEG hyperscanning research^79,80^, as well as with other brain signals and techniques, such the hemodynamic activity using fNIRS^45,81^.

### Statistical analysis

Two sets of analyses were performed concerning EEG dependent measures.

A first step of analysis included the application of coherence analysis for each dyad. We reported for this first step the mean trend of the coherence index for each dyad of participants.

A second step of analysis was applied to these coherence values considered as dependent measures of repeated measures ANOVAs with independent within factors Condition (2: focus on the breath, no focus on the breath) × Task (2: motor and cognitive) × Electrode (15: Fp1, Fp2, AFF5h, Fz, AFF6h, T7, C3, Cz, C4, T8, P3, Pz, P4, O1, O2). ANOVA was applied independently for each frequency band, for a total of four repeated measures ANOVAs by using SPSS (IBM SPSS Statistics, version 25).

For all ANOVA tests, in case of significant effects, pairwise comparisons were conducted to explore the significant interactions between simple effects, and the Bonferroni correction was applied to lessen the possible bias of repeated comparisons. The degrees of freedom for all ANOVA tests were adjusted using the Greenhouse–Geisser epsilon where required. Using partial eta squared (η^2^) indices, the magnitude of statistically significant effects was calculated.

## Supplementary Information

Supplementary Information.

## Data Availability

The datasets generated and analyzed for this study are available from the corresponding author on reasonable request.

## References

[1] Khalsa SS (2018). Interoception and mental health: A roadmap. Biol. Psychiatry Cogn. Neurosci. Neuroimaging.

[2] Schulz SM (2016). Neural correlates of heart-focused interoception: A functional magnetic resonance imaging meta-analysis. Philos. Trans. R. Soc. B Biol. Sci..

[3] Tsakiris M, De Preester H (2018). The Interoceptive Mind: From Homeostasis to Awareness.

[4] Weng HY (2021). Interventions and manipulations of interoception. Trends Neurosci..

[5] Palmer CE, Tsakiris M (2018). Going at the heart of social cognition: Is there a role for interoception in self-other distinction?. Curr. Opin. Psychol..

[6] Gao Q, Ping X, Chen W (2019). Body influences on social cognition through interoception. Front. Psychol..

[7] Arnold AJ, Winkielman P, Dobkins K (2019). Interoception and social connection. Front. Psychol..

[8] Burleson MH, Quigley KS (2021). Social interoception and social allostasis through touch: Legacy of the Somatovisceral Afference Model of Emotion. Soc. Neurosci..

[9] Balconi M, Angioletti L (2021). One’s interoception affects the representation of seeing others’ pain: A randomized controlled qEEG study. Pain Res. Manag..

[10] Balconi M, Angioletti L (2021). Interoception as a social alarm amplification system. What multimethod (EEG-fNIRS) integrated measures can tell us about interoception and empathy for pain?. Neuropsychol. Trends.

[11] Filippetti ML (2021). Being in tune with your body: The emergence of interoceptive processing through caregiver-infant feeding interactions. Child Dev. Perspect..

[12] Feldman R (2006). From biological rhythms to social rhythms: Physiological precursors of mother-infant synchrony. Dev. Psychol..

[13] Fotopoulou A, Tsakiris M (2017). Mentalizing homeostasis: The social origins of interoceptive inference. Neuropsychoanalysis.

[14] Atzil S, Gao W, Fradkin I, Barrett LF (2018). Growing a social brain. Nat. Hum. Behav..

[15] Balconi M, Angioletti L (2022). Interoceptive attentiveness induces significantly more PFC activation during a synchronized linguistic task compared to a motor task as revealed by functional Near-Infrared Spectroscopy. Brain Sci..

[16] Angioletti L, Balconi M (2022). Delta-Alpha EEG pattern reflects the interoceptive focus effect on interpersonal motor synchronization. Front. Neuroergonomics.

[17] Angioletti L, Balconi M (2022). The Increasing effect of interoception on brain frontal responsiveness during a socially framed motor synchronization task. Front. Hum. Neurosci..

[18] Angioletti L, Balconi M (2022). EEG brain oscillations are modulated by interoception in response to a synchronized motor vs. cognitive task. Front. Neuroanat..

[19] Boyadzhieva A, Kayhan E (2021). Keeping the breath in mind: Respiration, neural oscillations, and the free energy principle. Front. Neurosci..

[20] Allen M, Varga S, Heck DH (2022). Respiratory rhythms of the predictive mind. Psychol. Rev..

[21] Hammer M, Schwale C, Brankačk J, Draguhn A, Tort ABL (2021). Theta-gamma coupling during REM sleep depends on breathing rate. Sleep.

[22] Hsu SM, Tseng CH, Hsieh CH, Hsieh CW (2020). Slow-paced inspiration regularizes alpha phase dynamics in the human brain. J. Neurophysiol..

[23] Tort ABL, Brankačk J, Draguhn A (2018). Respiration-entrained brain rhythms are global but often overlooked. Trends Neurosci..

[24] Herrero JL, Khuvis S, Yeagle E, Cerf M, Mehta AD (2018). Breathing above the brain stem: Volitional control and attentional modulation in humans. J. Neurophysiol..

[25] Varga S, Heck DH (2017). Rhythms of the body, rhythms of the brain: Respiration, neural oscillations, and embodied cognition. Conscious. Cogn..

[26] Tschacher W, Meier D (2020). Physiological synchrony in psychotherapy sessions. Psychother. Res..

[27] Coomans E (2021). Intersubject EEG coherence in healthy dyads during individual and joint mindful breathing exercise: An EEG-based experimental hyperscanning study. Adv. Cogn. Psychol..

[28] Marzoratti A, Evans TM (2022). Measurement of interpersonal physiological synchrony in dyads: A review of timing parameters used in the literature. Cogn. Affect. Behav. Neurosci..

[29] Sänger J, Müller V, Lindenberger U (2012). Intra- and interbrain synchronization and network properties when playing guitar in duets. Front. Hum. Neurosci..

[30] Tei S (2009). Meditators and non-meditators: EEG source imaging during resting. Brain Topogr..

[31] Kubota Y (2001). Frontal midline theta rhythm is correlated with cardiac autonomic activities during the performance of an attention demanding meditation procedure. Cogn. Brain Res..

[32] Farb N (2015). Interoception, contemplative practice, and health. Front. Psychol..

[33] Tang YY, Tang R, Rothbart MK, Posner MI (2019). Frontal theta activity and white matter plasticity following mindfulness meditation. Curr. Opin. Psychol..

[34] Tang YY, Hölzel BK, Posner MI (2015). The neuroscience of mindfulness meditation. Nat. Rev. Neurosci..

[35] Cavanagh JF, Frank MJ (2014). Frontal theta as a mechanism for cognitive control. Trends Cogn. Sci..

[36] Yun K, Watanabe K, Shimojo S (2012). Interpersonal body and neural synchronization as a marker of implicit social interaction. Sci. Rep..

[37] Dumas G, Nadel J, Soussignan R, Martinerie J, Garnero L (2010). Inter-brain synchronization during social interaction. PLoS ONE.

[38] Meijer D, te Woerd E, Praamstra P (2016). Timing of beta oscillatory synchronization and temporal prediction of upcoming stimuli. Neuroimage.

[39] Tan H, Wade C, Brown P (2016). Post-movement beta activity in sensorimotor cortex indexes confidence in the estimations from internal models. J. Neurosci..

[40] Sedley W (2016). Neural signatures of perceptual inference. Elife.

[41] Morillon B, Arnal LH, Schroeder CE, Keitel A (2019). Prominence of delta oscillatory rhythms in the motor cortex and their relevance for auditory and speech perception. Neurosci. Biobehav. Rev..

[42] Cannon EN (2014). Action experience, more than observation, influences mu rhythm desynchronization. PLoS ONE.

[43] Quandt LC, Marshall PJ (2014). The effect of action experience on sensorimotor EEG rhythms during action observation. Neuropsychologia.

[44] Montague PR (2002). Hyperscanning: Simultaneous fMRI during linked social interactions. Neuroimage.

[45] Balconi M, Pezard L, Nandrino J-L, Vanutelli ME (2017). Two is better than one: The effects of strategic cooperation on intra- and inter-brain connectivity by fNIRS. PLoS ONE.

[46] Crivelli D, Balconi M (2017). Near-infrared spectroscopy applied to complex systems and human hyperscanning networking. Appl. Sci..

[47] Balconi M, Vanutelli ME (2017). Cooperation and competition with hyperscanning methods: Review and future application to emotion domain. Front. Comput. Neurosci..

[48] Novembre G, Sammler D, Keller PE (2016). Neural alpha oscillations index the balance between self-other integration and segregation in real-time joint action. Neuropsychologia.

[49] Kelsen BA, Sumich A, Kasabov N, Liang SHY, Wang GY (2022). What has social neuroscience learned from hyperscanning studies of spoken communication? A systematic review. Neurosci. Biobehav. Rev..

[50] Balconi M, Vanutelli ME (2018). EEG hyperscanning and behavioral synchronization during a joint action. Neuropsychol. Trends.

[51] Venturella I, Gatti L, Vanutelli ME, Balconi M (2017). When brains dialogue by synchronized or unsynchronized languages Hyperscanning applications to neuromanagement. Neuropsychol. Trends.

[52] Balconi M, Venturella I, Fronda G, Vanutelli ME (2020). Leader-employee emotional “interpersonal tuning”. An EEG coherence study. Soc. Neurosci..

[53] Balconi M, Fronda G, Cassioli F, Crivelli D (2022). Face-to-face vs. remote digital settings in job assessment interviews: A multilevel hyperscanning protocol for the investigation of interpersonal attunement. PLoS ONE.

[54] Balconi M, Cassioli F (2022). “We will be in touch”. A neuroscientific assessment of remote vs. face-to-face job interviews via EEG hyperscanning. Soc. Neurosci..

[55] Tripathi, V., Bhasker, L., Kharya, C., Bhatia, M. & Kochupillai, V. Electroencephalographic dynamics of rhythmic breath-based meditation. *bioRxiv.*10.1101/2022.03.09.483685 (2022).

[56] Lindenberger U, Li SC, Gruber W, Müller V (2009). Brains swinging in concert: Cortical phase synchronization while playing guitar. BMC Neurosci..

[57] Baker SN (2007). Oscillatory interactions between sensorimotor cortex and the periphery. Curr. Opin. Neurobiol..

[58] McKay LC, Evans KC, Frackowiak RSJ, Corfield DR (2003). Neural correlates of voluntary breathing in humans. J. Appl. Physiol..

[59] Pérez A, Carreiras M, Duñabeitia JA (2017). Brain-To-brain entrainment: EEG interbrain synchronization while speaking and listening. Sci. Rep..

[60] Aftanas LI (1998). Non-linear dynamic complexity of the human EEG during evoked emotions. Int. J. Psychophysiol..

[61] Beauregard M, Courtemanche J, Paquette V (2009). Brain activity in near-death experiencers during a meditative state. Resuscitation.

[62] Aftanas LI, Golocheikine SA (2001). Human anterior and frontal midline theta and lower alpha reflect emotionally positive state and internalized attention: High-resolution EEG investigation of meditation. Neurosci. Lett..

[63] Takahashi T (2005). Changes in EEG and autonomic nervous activity during meditation and their association with personality traits. Int. J. Psychophysiol..

[64] Matthews SC, Paulus MP, Simmons AN, Nelesen RA, Dimsdale JE (2004). Functional subdivisions within anterior cingulate cortex and their relationship to autonomic nervous system function. Neuroimage.

[65] Tang Y-Y (2009). Central and autonomic nervous system interaction is altered by short-term meditation. Proc. Natl. Acad. Sci. U. S. A..

[66] Pfurtscheller G (1981). Central beta rhythm during sensorimotor activities in man. Electroencephalogr. Clin. Neurophysiol..

[67] Salmelin R, Hämäläinen M, Kajola M, Hari R (1995). Functional segregation of movement-related rhythmic activity in the human brain. Neuroimage.

[68] McFarland DJ, Miner LA, Vaughan TM, Wolpaw JR (2000). Mu and beta rhythm topographies during motor imagery and actual movements. Brain Topogr..

[69] Neuper C, Pfurtscheller G (2001). Evidence for distinct beta resonance frequencies in human EEG related to specific sensorimotor cortical areas. Clin. Neurophysiol..

[70] Gong A (2021). A Review of neurofeedback training for improving sport performance from the perspective of user experience. Front. Neurosci..

[71] Samuel, R. D. & Brom, D. Potential applications of somatic experiencing® in applied sport psychology. *J. Sport Psychol. Action* 1–13 (2022).

[72] Rivest-Gadbois E, Boudrias MH (2019). What are the known effects of yoga on the brain in relation to motor performances, body awareness and pain? A narrative review. Complement. Ther. Med..

[73] Jelinčić V, Van Diest I, Torta DM, von Leupoldt A (2022). The breathing brain: The potential of neural oscillations for the understanding of respiratory perception in health and disease. Psychophysiology.

[74] Farb NAS, Segal ZV, Anderson AK (2013). Attentional modulation of primary interoceptive and exteroceptive cortices. Cereb. Cortex.

[75] Kawasaki M, Yamada Y, Ushiku Y, Miyauchi E, Yamaguchi Y (2013). Inter-brain synchronization during coordination of speech rhythm in human-to-human social interaction. Sci. Rep..

[76] Oostenveld R, Praamstra P (2001). The five percent electrode system for high-resolution EEG and ERP measurements. Clin. Neurophysiol..

[77] Ludwig A, Miriani RM, Langhals NB, Joseph MD, David J (2008). Using a common average reference to improve cortical neuron recordings from microelectrode arrays. J. Neurophysiol..

[78] Wheland, D. *et al.* Robust identification of partial-correlation based networks with applications to cortical thickness data. In *Proceedings—International Symposium on Biomedical Imaging* 1551–1554. 10.1109/ISBI.2012.6235869 (2012). 10.1109/ISBI.2012.6235869 PMC4705556 26753056

[79] Balconi M, Gatti L, Vanutelli ME (2018). EEG functional connectivity and brain-to-brain coupling in failing cognitive strategies. Conscious. Cogn..

[80] Balconi M, Vanutelli ME (2018). Functional EEG connectivity during competition. BMC Neurosci..

[81] Balconi M, Vanutelli ME, Gatti L (2018). Functional brain connectivity when cooperation fails. Brain Cogn..

