# Supplementary material for: Dyadic inter-brain EEG coherence induced by interoceptive hyperscanning
Source: Sci Rep. 2023 Mar 16;13:4344. doi: 10.1038/s41598-023-31494-y (PMC10020471; doi:10.1038/s41598-023-31494-y)
Supplement: Supplementary file 1 — Supplementary Information. [file 41598_2023_31494_MOESM1_ESM.docx]

**Supplementary Material**

Table 1. Self-report data collected during the debriefing phase, at the end of the tasks, in which participants declared (on a scale from 0 to 10 points) the attention they paid to their breathing, to the other person and to the task. Data are shown as mean and ± standard deviation.

|  | **Motor synchronization task** | | **Sign.** | **Cognitive synchronization task** | | **Sign.** |
| --- | --- | --- | --- | --- | --- | --- |
|  | **Focus condition** | **No-focus condition** |  | **Focus condition** | **No-focus condition** |  |
| **Attention to breath** | 9.16±0.89 | 5.88±1.09 | **< .001** | 8.33±1.10 | 5.61±2.26 | **< .001** |
| **Attention to the task** | 6.05±1.39 | 6.05±1.07 | n.s. | 6.00±1.33 | 5.77±2.01 | n.s. |
| **Attention to the other** | 6.11±1.19 | 6.00±1.33 | n.s. | 5.77±1.47 | 5.35±1.67 | n.s. |
